# Balancing Performance and Health in Elite Hungarian Athletes: The Relationship Among Disordered Eating Risk, Body Composition, and Nutrition Knowledge

**DOI:** 10.3390/nu17020231

**Published:** 2025-01-09

**Authors:** Réka Erika Kovács, Merve Alpay, István Karsai, Gusztáv József Tornóczky, Andrea Petróczi, Szilvia Boros

**Affiliations:** 1 Doctoral School of Education, Faculty of Pedagogy and Psychology, ELTE Eötvös Loránd University, 1075 Budapest, Hungary; rekaerika@student.elte.hu; 2 National Institute for Sports Medicine, H-1123 Budapest, Hungary; 3 Institute of Health Sciences, Faculty of Sport Science, Osmaniye Korkut Ata University, Osmaniye 8000, Turkey; merve.alpay@windowslive.com; 4 Physical Education and Exercise Centre, Medical School, University of Pecs, H-7624 Pecs, Hungary; istvan.karsai@aok.pte.hu; 5 Institute of Health Promotion and Sport Sciences, Faculty of Education and Psychology, ELTE Eötvös Loránd University, H-1053 Budapest, Hungary; gjtornoczky@gmail.com (G.J.T.); a.petroczi@kingston.ac.uk (A.P.); 6 Department of Applied and Human Sciences, Faculty of Health, Science, Social Care and Education, Kingston University, London KT1 2EE, UK; 7 Department of Health and Nursing Sciences, Faculty of Health and Sport Sciences Széchenyi István University, H-9026 Gyor, Hungary

**Keywords:** disordered eating, elite athletes, nutrition knowledge, body composition, EAT-26, DESA-6H

## Abstract

Background: disordered eating (DE) and eating disorders (ED) can negatively impact athletes’ health, wellbeing, and athletic performance. Objective: this cross-sectional study aims to assess DE risk, body composition, and nutrition knowledge among elite Hungarian athletes. Methods: DE risk was assessed using DESA-6H and EAT-26 scales, nutrition knowledge through the Abridged Nutrition for Sport Knowledge Questionnaire (A-NSKQ), and body composition with the OMRON BF511 device. The data were analyzed using Kendall’s tau correlations, Mann–Whitney U tests, and ROC analysis. Results: a total of 71 athletes participated (39.4% males, mean age = 24.8 years, SD = 4.8 years and 60.6% females, mean age = 24.3 years, SD = 4.3 years). At-risk scores on the DESA-6H scale were recorded for nine athletes (12.7%), while 32.4% scored in the risk zone on the EAT-26, with female athletes in aesthetic, endurance and weight-dependent sports being most affected. Low BF was observed in four males and four females. Nutrition knowledge (49.1%) was below the acceptable threshold. DESA-6H significantly correlated with EAT-26 scores, BMI, sports nutrition knowledge, and A-NSKQ total scores. A statistically significant difference by gender was found in the EAT-26 total score (*p* = 0.019, d = 0.65). Risk groups significantly differed in A-NSKQ scores (*p* = 0.026, d = 0.511) and sport nutrition knowledge, specifically (*p* = 0.016, d = 0.491). Using EAT-26 to identify at-risk athletes and the DESA-6H recommended cut-off, the ROC analysis showed a sensitivity of 29.1% and a specificity of 95.7%. Conclusions: insufficient nutrition knowledge plays a role in being at-risk for DE and ED. These results underscore the need for early detection, early sport nutrition education across all elite athletes, with particular attention to female athletes in aesthetic, endurance and weight-dependent sports, and for monitoring these athletes to prevent DE. Further work is warranted to optimize screening tools such as EAT-26 and DESA-6H for elite athletes.

## 1. Introduction

Optimal, individualized nutrition is essential for athletes, supporting effective training adaptation, recovery, favorable body composition, and overall health and performance [1,2]. Nutrition strategies developed by dietitians and sport nutritionists are crucial for health promotion, body composition optimization, performance enhancement, and even post-injury recovery [3,4,5]. However, many athletes rely on unqualified sources—such as coaches, parents, and teammates—for nutritional advice, an approach which can lead to unsupervised practices that heighten the risk of disordered eating (DE) and eating disorders (ED) [6]. DE and ED are reported at higher rates among athletes compared to the general population. In 2019, the International Olympic Committee (IOC) noted that 6–45% of female athletes and 0–19% of male athletes were affected [5].

DE and ED are prevalent across various sport disciplines, with a heightened risk observed in aesthetic, endurance, and weight-dependent sports [7,8]. Notably, team and strength sports also show a considerable prevalence [8,9,10]. According to Mancine and colleagues, sport disciplines are distinguished as lean sports and non-lean sports, with three subgroups each, based on the emphasis on low body weight. The risk group consists of lean athletes and includes aesthetic (e.g., dance, gymnastics, rhythmic gymnastics), endurance (e.g., long-distance running, swimming, diving), and weight-dependent sports (e.g., judo, karate, boxing). The latter includes ball games, strength sports, and endurance sports [8]. The literature does not differentiate between DE and ED in terms of risk groups, but Martinsen and Sundgot-Borgen created another classification system [7], where fifty sports were classified as weight-sensitive and less weight-sensitive, for a total of eight subgroups. Weight-sensitive sports include aesthetic (e.g., dance, gymnastics, etc.), weight-dependent (e.g., judo, karate, taekwondo, etc.), some technical (e.g., athletics jumping events), and endurance (e.g., middle- and long-distance running, rowing, cycling, etc.) sports. The less weight-sensitive group includes ball games (e.g., basketball, handball, tennis, volleyball, etc.), strength sports (e.g., sprint running), some technical (e.g., golf, shooting sports, chess, fencing, etc.) and heavyweight (e.g., hammer and discus throwing, javelin throwing, etc.) sports [7]. Participants in weight-sensitive sports have been found to be at higher risk, regardless of gender [11,12,13].

Strict eating patterns, combined with high intensity and duration of exercise, contribute to the development of eating disorders and may also delay puberty in younger athletes [14,15,16]. DE and ED appear to form a continuum, often beginning with weight loss concerns and progressing from DE behaviors to clinical ED [17]. According to the American Psychiatric Association (APA), this continuum spans from an intense preoccupation with weight and food to restrictive eating behaviors and, ultimately, diagnosable ED [18]. DE encompasses a range of pathological behaviors aimed at weight reduction or performance enhancement, including restrictive eating, bingeing, and purging, wearing excess clothing to induce sweating, and using saunas or weight-loss supplements [19]. EDs, in contrast, are characterized by a pervasive focus on food that disrupts daily life. Behaviors such as binge eating and purging may occur multiple times weekly, with added, unscheduled exercise performed to burn additional calories [19].

Even without an ED diagnosis, DE can significantly impair health due to low energy availability (LEA), nutrient deficiencies, and low body fat (BF) [20,21,22]. Limited longitudinal research exists on the progression from DE to ED, yet current findings suggest that DE behaviors are unlikely to resolve without intervention and may progress to ED over time [23,24,25].

Development of DE and ED is influenced by several factors, including psychological and physical stressors (e.g., early specialized training and increased load), individual traits (e.g., genetics, personality), family history (e.g., previous EDs or mood disorders), and sociocultural factors such as gender, age, social pressure, and nutrition knowledge [26,27]. Concerning diagnostic criteria, according to the Diagnostic and Statistical Manual of Mental Disorders (DSM-V), AN and bulimia nervosa (BN), as well as the binge eating disorder (BED), can be classified as three main EDs. AN was first described by Richard Morton in 1689, and became an independent disorder in 1873 [28]. BN was described by Russel in 1979 and was included in the DSM-III criteria the following year [29]. Finally, BED was elevated to an independent disorder in 1991 and later became part of the DSM-IV system [29,30]. Although not classified as a major ED, muscle dysmorphia or inverse AN is important to mention among athletes and is considered a subtype of body dysmorphic disorder (BDD) in the DSM-V [31,32]. The preoccupation with muscularity pushes these individuals to adopt a restricted calorie intake, train excessively, and consume anabolic steroids [33]. Although the diagnosis of DE and ED falls under the expertise of medical professionals (e.g., internists, neurologists, family doctors, psychiatrists) and clinical psychologists [34], other healthcare professionals also play a crucial role in the prevention and early detection of DE and ED among athletes [35]. Coaches, who spend extensive time with athletes, also play a key role in shaping attitudes toward body image and nutrition, underscoring the need to equip them with the knowledge to provide accurate guidance and refer athletes to professionals when necessary [36,37]. In response, several questionnaires have been developed to assess nutrition knowledge in athletes and coaches; however, studies show that knowledge in both groups requires improvement [38,39,40].

Given the impact of DE and ED on athletes’ health, performance, and long-term well-being, the need for understanding DE risk factors is evident. DE is linked to serious physiological and psychological consequences, such as compromised immunity, reduced bone density, cognitive impairments, and decreased mental well-being, all of which can impair athletic performance and heighten the risk of career-ending injuries [15]. Research further suggests that DE behaviors can escalate to ED without timely intervention [17]. Despite this vulnerability, athletes often lack adequate sport-specific nutrition knowledge and may rely on unqualified sources for dietary advice, a phenomenon which can contribute to unsupervised practices and an increased DE risk [6].

Despite the global significance of disordered eating (DE) and eating disorders (ED), Hungarian studies on this topic—with two exceptions—are lacking. A 2009 survey of 72 elite athletes across various sports reported anorexia nervosa in 16.7% and bulimia nervosa in 6.9% of the participants [41]. In 2024, Keczeli et al. [42] studied ED prevalence among 222 competitive rowers, where lightweight rowers scored significantly higher on the perfectionism and interoceptive awareness subscales of the Eating Disorder Inventory questionnaire (EDE) compared to open-weight rowers, but no athletes were classified as having an ED. Therefore, the primary aim of this study is to address this hiatus by examining DE risk, body composition, and nutrition knowledge among elite Hungarian athletes, a population with limited research representation. By analyzing these factors within a Hungarian context, this study can enrich the literature and guide culturally relevant strategies for preventing, detecting, and intervening in cases of DE and ED. Identifying sports, demographics, and nutrition knowledge levels associated with a higher DE risk can also inform stakeholders—including sport nutritionists, coaches, and healthcare providers—in developing targeted support measures that promote health, optimize performance, and safeguard athletes’ long-term well-being. A secondary aim is to evaluate the diagnostic accuracy of the disordered eating screen for athletes (DESA-6H) [43,44] in Hungarian elite athletes.

## 2. Materials and Methods

### 2.1. Study Design

The study employed a cross-sectional quantitative design, utilizing a combination of survey-based, self-reported assessments alongside body composition measures. This design allowed for the simultaneous collection of data on disordered eating risk, nutrition knowledge, and body composition within a single time frame, providing a snapshot of these variables among elite Hungarian athletes. By integrating self-reported behavioral data with objective body measurements, the study aims to provide a comprehensive picture of disordered eating risk contextualized in nutrition knowledge and physical health markers.

### 2.2. Data Collection

Data collection for this study was conducted from 10 February to 31 July 2024, targeting elite athletes over 18 years of age. Recruitment took place at the National Institute for Sports Medicine in Budapest, Hungary, using convenience sampling. Posters featuring a QR code were displayed on-site to encourage participation. By scanning the QR code, athletes could access the study information, including the study’s purpose, completion guidelines, and data security measures. Once the athletes had reviewed and accepted the study conditions (by checking a consent box), they were directed to complete the following questionnaires: the Hungarian version of the disordered eating screen for athletes (DESA-6H), the Eating Attitudes Test (EAT-26), and the Abridged Nutrition for Sport Knowledge Questionnaire (A-NSKQ) [44,45,46]. In addition, participants provided sociodemographic details (e.g., sex, age) and sport-related information (e.g., sport discipline, years in sport, training hours per week). Answering a total of 94 questions took an average of 27 (SD = 10) minutes. It is similar to a previous study by Trakman et al. (2018), where 89 questions took 25 min to answer [45,46]. After completing the survey, the participants registered at the outpatient dietetic care for physical measurements, including height measured with a Seca 200 device and body composition measured with an OMRON BF511 body composition measurement device, where their body weight, BMI, and body fat percentage (PBF) were recorded. Each athlete received an individual code to ensure anonymity. Data responses collected via Qualtrics were exported to Microsoft Excel 2007 for analysis.

### 2.3. Measures 

#### 2.3.1. DESA-6—Disordered Eating Screen for Athletes

The DESA-6 questionnaire was first tested in 2017 on adult triathletes [36]. Initially unnamed, the tool focused on “competitive athlete factors”, with six questions hypothesized to correlate with a heightened risk of disordered eating compared to “triathlon-specific factors”. With a sample size of 1033 participants, the findings confirmed that competitive athlete factors were indeed associated with a higher risk of DE, while no correlation was found with triathlon-specific factors [38]. The six questions entailed different ratings for each question, from −1 to 2, covered varying response options, ranging from two to five choices, and assessed topics such as injury frequency, fear of weight gain, body weight satisfaction, ideal body weight for peak performance, diet habits, and weight-related comments. Scores ranged from 0 to 9, with a cut-off score of ≥3 indicating a DE risk [43]. The test–retest reliability (r) was 0.83 and the DESA-6 scores were positively correlated with measures of eating pathology [43]. The Hungarian version, DESA-6H, was used in this study with permission [44]. The Guttman split-half coefficient was below the acceptable level (λ = 0.40).

#### 2.3.2. EAT-26—Eating Attitudes Test

The EAT-26, an adaptation of the original 40-item EAT [47,48], was designed to screen for anorexia-related behaviors and to serve as a prognostic tool [48]. Now widely used as a self-report screening tool for eating attitudes, the EAT-26 uses a six-point Likert-type scale across three subscales: dieting behavior (13 questions, maximum points: 39), oral control (six questions, maximum points: 18), and tendencies toward bulimia and food preoccupation (six questions, maximum points: 18) [49]. For this study, the Hungarian version [50,51] was employed and a cut-off score of ≥12 was used [43]. The internal consistency reliability of the total questionnaire and dieting behavior subscale was acceptable (expressed as Cronbach’s alpha) at 0.78 and 0.74, respectively. However, the internal consistecy reliability of the bulimia subscale was questionable (α = 0.66), and the oral control subscale was unacceptable (α = 0.46). The values below the acceptable level are probably due to the fact that, in the bulimia and oral control subscales, we also found one item that showed a negative correlation: item 2 (oral control) and item 26 (bulimia). Further analyses were implemented with the values of the entire questionnaire.

#### 2.3.3. A-NSKQ—Abridged Nutrition for Sport Knowledge Questionnaire

While various nutrition knowledge questionnaires exist [52], the A-NSKQ is among the few that assess both general and sport-specific nutrition knowledge and can be applied across different sports [45,46]. Originally developed as the NSKQ in 2017 with 89 items [46], a shortened version was released in 2018 to improve completion rates [45]. The A-NSKQ comprises 35 questions, split between general (11 items) and sport-specific (24 items) nutrition topics, covering weight management, macronutrients, micronutrients, supplements, sport nutrition, and alcohol. Responses are scored as one point per correct answer, with a maximum score of 35 [45]. The Hungarian translation, used with permission from the original authors, was validated for this study with a reasonable internal consistency reliability in the case of the total questionnaire (KR-20 = 0.76) and sports nutrition knowledge subscale (KR-20 = 0.70), but poor internal consistency reliability in the general nutrition knowledge subscale (KR-20 = 0.52).

### 2.4. Ethical Considerations

This study was conducted following ethical standards and received approval from the first author’s institutional ethics board (ELTE PPK KEB 2024/10). Informed consent was obtained from all participants, who were fully briefed on the study’s purpose, procedures, and their right to withdraw at any time without consequences. Participation was voluntary, and athletes received no financial compensation; however, their body composition measurements were shared with them as part of their health information. After the data collection was completed, data were anonymized using individual codes to protect the participants’ identity, with all digital data securely stored on password-protected systems accessible only to authorized research personnel.

To mitigate against potential psychological distress, participants were provided with information on resources for mental health and nutrition counseling if needed. Participants were informed that, if the assessment identified an elevated risk of DE, they would be referred to appropriate support resources for follow-up care upon request.

### 2.5. Data Analysis

Descriptive statistics, including mean (M), standard deviation (SD), and frequency (%), were used when presenting details on gender, age, BMI, PBF, at-risk and non-risk groups, scores of DESA-6H, EAT-26, and A-NSKQ. The normal distribution assumption was tested with the Shapiro–Wilk test. The possible relationship between DESA-6H and EAT-26 total scores, in addition to EAT-26 subscales with BMI, PBF, general and sport nutrition knowledge, and total nutrition knowledge score, was examined using Kendall’s tau correlation tests. Non-parametric Mann–Whitney U tests were used to investigate pairwise differences between the following variables: DESA-6H and EAT-26 scores by gender and nutritional status, at-risk and non-risk groups based on DESA-6H and EAT-26 scores in nutrition knowledge. Effect sizes were determined using Cohen-*d* effect size indicators [53,54] and interpreted for Mann–Whitney U tests, as follows: d < 0.3 (small effect), 0.3 ≤ d < 0.5 (medium effect), d ≥ 0.5 (large effect). Cohen’s κ was run to determine if there was agreement between DESA-6H and EAT-26 categorizations, and whether certain athletes were at risk or not was interpreted as follows: 0.01–0.20 none to slight, 0.21–0.40 fair, 0.41–0.60 moderate, 0.61–0.80 substantial, and 0.81–1.00 almost perfect agreement [55,56]. To explore the diagnostic accuracy of DESA-6H with respect to how well the test separated DE risk groups among elite Hungarian athletes, ROC (receiver operating characteristic) analysis and area under the curve (AUC) were used with Youden’s index (*J*) to determine an optimal cut-off value. For this analysis, the at-risk status was determined by the EAT-26, a tool which has been widely used in sport-related settings and beyond to screen for ED risks [57,58]. In the statistical analyses, *p* < 0.05 was considered statistically significant. All calculations were conducted using IBM SPSS 29.0.

## 3. Results

Of the 87 athletes who registered for the study, 16 were excluded because they were minors. The final sample included 71 athletes (28 males and 43 females), with an average age of 23.8 (SD = 4.2) years (24.2 (SD = 2) years for men and 23.6 (SD = 4) years for women). Participants represented 28 different sport disciplines, categorized as aesthetic (*n* = 7), endurance (*n* = 14), weight-dependent (*n* = 6), strength (*n* = 2), strength–endurance (*n* = 15), team (*n* = 16), and technical sports (*n* = 11). Aesthetic sports included skydiving, dance, and rhythmic gymnastics, while endurance sports encompassed orienteering, pentathlon, racewalking, swimming, and cycling. Weight-dependent athletes participated in boxing, karate, judo, taekwondo, and wrestling, and strength athletes included discus throwers and hammer throwers. Strength–endurance sports comprised rowing, canoeing, javelin throw, long jump, and hurdles. Team sport athletes represented handball, water polo, and volleyball, while technical sport participants included fencing, shooting, archery, chess, and sailing. Participants reported an average of 14.1 (SD = 4) years of practice in their respective sports (14 (3.5) years for men and 14.2 (SD = 5) years for women). The average weekly training load was 18.4 (SD = 7.8) hours for men and 17.9 (SD = 7.3) hours for women.

### 3.1. DESA-6H and EAT-26 Scores

**Figure 1:**
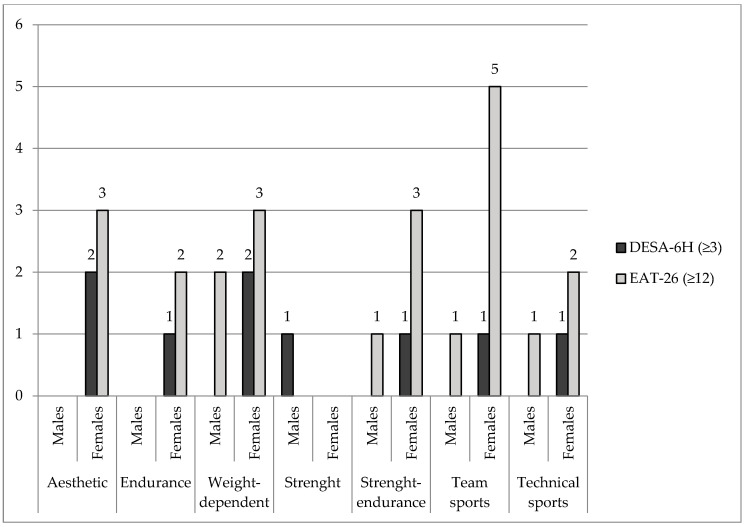
Athletes classified under the risk group from different sport disciplines based on DESA-6H and EAT-26 scores (*n* = 71).

The mean DESA-6H score across the sample was 0.7 (SD = 1.6), with women scoring slightly higher 0.8 (SD = 1.9) than men 0.5 (SD = 1.1). Based on DESA-6H scores, nine athletes (one male and eight females) were assigned to the risk group for disordered eating. For the EAT-26, the mean score in the total sample was 11.2 (SD = 7.0), with men averaging at 8.5 (SD = 4.2) and women at 12.9 (SD = 8.0). Overall, 23 athletes (five males and 18 females) scored in the risk zone for eating disorders. Figure 1 shows the characteristics of the athletes classified in the risk group based on DESA-6H and EAT-26 scores.

The most affected athletes were female team athletes (*n* = 5) and those in aesthetic, endurance, and weight-dependent sports (*n* = 3 in each group).

For EAT-26, we examined the individual factors as well. In case of dieting behavior, scores ranged between 0 and17, with 2 (*n* = 16, 22.5%), 0 (*n* = 11, 15.4%), and 8 (*n* = 6, 8.4%) being the most frequent in the sample. In the oral control subscale, athletes scored between 0 and 10, but over half scored 0 (*n* = 17, 24%), 2 (*n* = 16, 22.5%), or 3 (*n* = 13, 18.3%). Finally, examining bulimia and the food preoccupation scale, scores ranged from 2 to 14, with the most frequent score being 3 (*n* = 51, 71.8%), 4 (*n* = 4, 5.6%), or 9 (*n* = 3, 4.2%).

### 3.2. Body Composition

Body composition classifications were based on the reference ranges provided by the World Health Organization (WHO) and the American Council on Exercise (ACE) [59,60]. Most athletes (74%) fell within the normal BMI range. One male swimmer and two females (one orienteer and one dancer) were classified as underweight, while one male and one female racewalker approached the lower limit of the normal range (18.7 and 18.6 kg/m^2^, respectively). Four male athletes (two shooters, one discus thrower, and one javelin thrower) and three female athletes (a shooter, a water polo player, and a canoeist) were classified as overweight. Regarding PBF, four males (one each from swimming, canoeing, dancing, and orienteering) had low BF, while others in shooting, archery, discus throwing, and volleyball showed high levels. Among female athletes, one athlete each from orienteering, pentathlon, rhythmic gymnastics, and long jump had a low PBF, while one water polo player exhibited high BF.

### 3.3. Nutrition Knowledge

**Figure 2:**
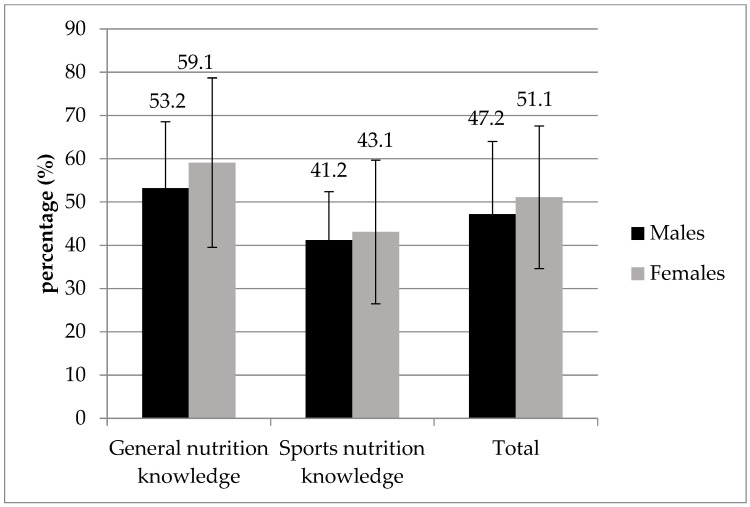
General and sports nutrition knowledge results.

On the A-NSKQ questionnaire, the whole sample scored an average of 56.1% in general nutrition knowledge and 42.1% in sports nutrition knowledge, resulting in an overall score of 49.1%. The greatest gender difference (with higher scores among women) was observed in relation to the general nutrition knowledge, while scores on sport-specific questions were nearly equivalent (Figure 2).

### 3.4. Relationship Among Disordered Eating Risk, Body Composition, and Nutrition Knowledge

The relationships between the measures used to assess athletes’ DE risks, body composition, and nutrition knowledge are shown in Table 1.

Regarding disordered eating risk, the DESA-6H significantly correlated with EAT-26 total score across the total sample. For body composition, a significant correlation was observed with DESA-6H and BMI, but no relationship was found between DESA-6H and PBF, nor between EAT-26 total scores and BMI or PBF. With respect to nutrition knowledge, DESA-6H showed significant correlations with sport nutrition knowledge and A-NSKQ total scores. Similar relationships were shown between EAT-26 scores and general-, sport nutrition knowledge, and A-NSKQ total scores, respectively. Statistically significant, strong, positive correlations were only found within the nutrition knowledge measures (0.51–0.85). Across domains, correlations ranged between 0.22 and 0.29, suggesting positive but weak relationships.

### 3.5. Differences Across Gender and Nutritional Status in Disordered Eating Risk and Eating Attitudes

Statistically significant differences with a large effect size were found in relation to the EAT-26 total scores of men and women, with females scoring higher than males. However, no significant differences showed between genders in the case of DESA6-H scores (Table 2).

When investigating the differences between nutritional status categories, we set three groups based on the BMI results (underweight, normal, overweight, and obese). Only one participant was categorized as obese (BMI = 33.4 kg/m^2^) and was added to the overweight group.

Due to the small sample size, a comparison among the three groups was not possible. Differences between the two groups (normal vs. overweight and obese), however, showed a statistically significant difference with a large effect size between the nutritional status and DESA-6H, with overweight and obese athletes scoring higher. There were no significant differences in the EAT-26 total scores. Our results are detailed in Table 3. For most variables, the overweight and obese group had less favorable scores.

### 3.6. Differences Across At-Risk and Non-Risk Groups Based on DESA-6H and EAT-26 Scores in Nutrition Knowledge

The number of participants identified as ‘at risk’ and ‘non-risk’ based on their DESA-6H scores were unbalanced (at-risk, *n* = 9, mean score: 4.11, SD = 0.92; non-risk, *n* = 62, mean score: 0.24, SD = 1.02)

No significant differences in nutrition knowledge and its factors were found between DESA-6H-based at-risk and non-risk groups (Table 4).

Differences in nutrition knowledge between the EAT-26 risk groups (at-risk, *n* = 47, mean score: 11.18 (SD = 7.02); non-risk, *n* = 24, mean score: 19.08 (SD = 6.48)) are presented in Table 5.

A statistically significant, medium-effect-size difference was found in relation to sport nutrition knowledge and a large-effect-size difference was found in relation to the nutrition knowledge total score; however, there was no difference in general nutrition knowledge between the EAT-26-based non-risk and at-risk groups.

### 3.7. At-Risk and Non-Risk Athlete Profiles

**Figure 3:**
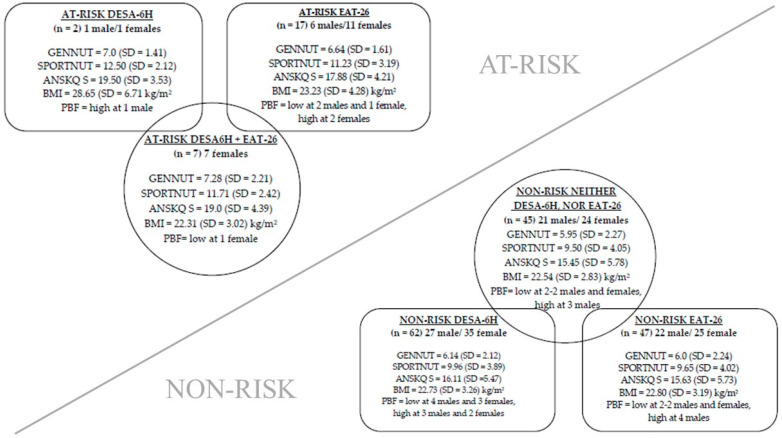
Overview of the athletes’ at-risk status of disordered eating, body composition, and nutrition knowledge. *n* = number of participants, SD = standard deviation, GENNUT: A-NSKQ general nutrition knowledge, SPORTNUT = A-NSKQ sport nutrition knowledge, ANSKQ S = A-NSKQ total score, BMI = body mass index, PBF = percent body fat.

To bring all the results together, Figure 3 depicts the observed profiles for at-risk and non-risk athletes.

As shown in Figure 3, 12.7% of the athletes was identified as ‘at-risk’ based on DESA6-H and 32.4% based on EAT-26, with men scoring less severely. There was a statistically significant fair agreement between the two variables, κ = 0.294, *p* = 0.03. For non-risk participants, a 63.3% overlap was found between DESA-6H and EAT-26, whilst only 24.03% of the at-risk athletes were identified by both screening tools. Thus, the remaining 12.67% was not categorized in the same group (at-risk or non-risk). Interestingly, during body composition measurements, there was not a large proportion of abnormal results, as only 4.2% of the athletes were underweight and 10% of them were overweight. Similarly, 11.2% had a low and 7.0% high body fat percentage. When assessing nutrition knowledge, females scored higher in both fields (general and sport nutrition knowledge); however, the average scores did not meet the acceptable level neither for males nor for females.

### 3.8. The Diagnostic Accuracy of DESA-6H to Separate DE Risk Groups

**Figure 4:**
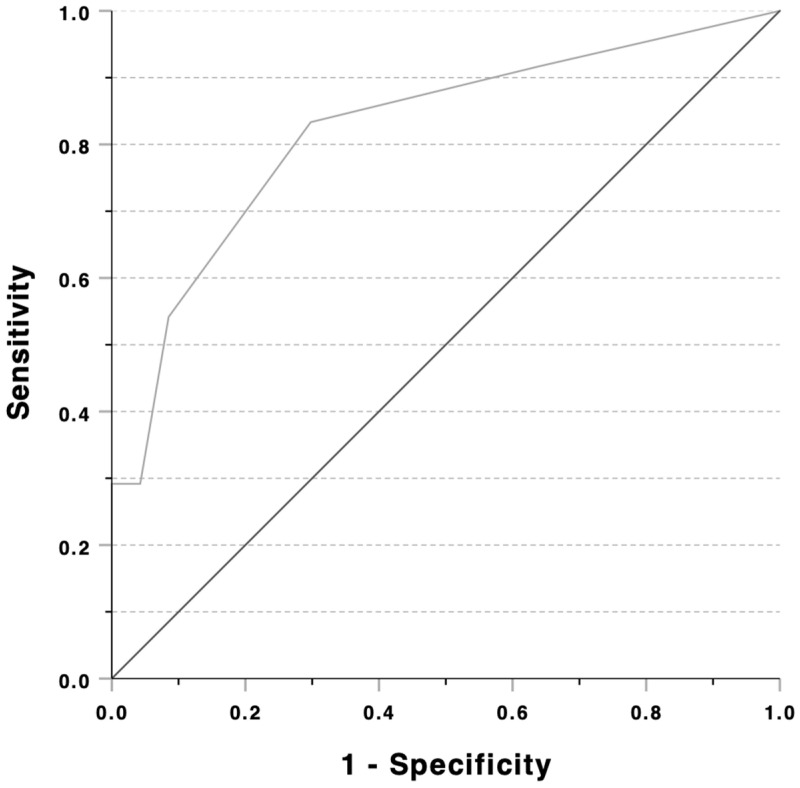
ROC curve analysis of the DESA-6H.

To establish evidence that DESA-6H is a good screening tool to distinguish between at-risk and non-risk groups, a ROC analysis was executed. At-risk and non-risk groups were based on EAT-26, where athletes with a score of 12 or higher were identified as being ‘at risk’. When examining the diagnostic value—accuracy of the test—for DESA-6H, the true positive rate (sensitivity) was measured against the false positive rate (1-specificity) with the ROC curve. In this present case, under the curve values (AUC = 0.82, 95%CI: 0.71–0.93, *p* < 0.001) showed that DESA-6H was able to identify at-risk athletes for disordered eating (Figure 4).

The Youden’s Index (*J*) is often used, as it provides the best balance between sensitivity and specificity. In our case, the DESA-6Hmax (*J* = 0.54) cut-off was set to 0.50, with a sensitivity of 83.3% and a specificity of 70.2%. According to international recommendations for DESA-6H, the cut-off point is ≥3 (*J* = 0.25), with a sensitivity of 29.1% and a specificity of 95.7%. In the present sample, we took this as a basis for the previously presented analyses. Table 6 presents the statistical values according to the two cut-off definitions in terms of separating the DE groups. Based on the two cut-off points, it can be seen that the international standard had a high specificity and a low sensitivity in our case. The best balance that we indicate has a higher sensitivity and a lower specificity compared to the international standard. However, it is worth noting that there was no meaningful difference in accuracy between the results of the analyses based on the two cut-off points.

## 4. Discussion

This study conducted a comprehensive assessment of 71 elite athletes, focusing on their nutrition knowledge and body composition while evaluating their risk status using two newly validated screening tools. The primary objective was to deepen the understanding of the relationship among DE risk, body composition, and nutrition knowledge. By examining these interconnected factors, the study aimed to uncover patterns and correlations that may inform both practice and policy in the context of athlete health management. The findings also provide valuable insights into the functionality and applicability of the two screening tools for disordered eating in athletes (DESA-6H) and eating disorders (EAT-26), respectively. Specifically, the study sheds light on how these tools differ or complement each other in identifying at-risk individuals, specifically athletes. As one of the screening tools is general (EAT-26) and the other is sport-specific (DESA-6H), our results offer a foundation for refining screening methodologies in future research in elite sport contexts as previous studies have also mentioned [61,62]. This dual focus not only advances knowledge in the field of sport nutrition and athlete well-being, but also contributes to the ongoing effort to develop more precise and effective diagnostic and intervention strategies.

### 4.1. At-Risk Athletes Across Gender and Sport Disciplines

The study revealed that female athletes, particularly those in team, aesthetic, endurance, and weight-dependent sports, were more likely to score in the risk zone for DE in both the DESA-6 and EAT-26 questionnaires. This is consistent with previous research that identifies female athletes as more vulnerable to DE behaviors due to increased pressures around body image and weight [63,64,65,66]. For example, prior studies have shown that female bodybuilders, gymnasts, and endurance athletes face a heightened risk of DE, with some even reaching clinical thresholds for eating disorders [67,68]. Cultural comparisons, such as the study by Okano et al. (2005) on Japanese and Chinese athletes, suggest that vulnerability to DE may also be shaped by sociocultural factors, with prevalence rates varying between countries and even among athletes in the same sport [69]. These findings emphasize the need for DE prevention strategies that address both sport-specific and cultural pressures. When comparing nutrition knowledge of at-risk and non-risk athletes, at-risk individuals were more informed around nutrition with large effect sizes for sports nutrition knowledge and the A-NSKQ total score. This result aligns with a systematic review which showed that athletes’ nutrition knowledge does not necessarily influence conscious food choices [40].

DESA-6 is a relatively new assessment tool for screening disordered eating risk; therefore, there is a limited number of studies implementing this screening tool for athletes. Although results, so far, have shown that DESA-6 is a reliable questionnaire [68], the recommended cut-off value for at-risk status could be challenged on practical grounds. Whilst the recommended score of 3 or higher leads to a high level of specificity, our results suggest that it may lead to missing athletes who could also be ‘at risk’ and in need of support and intervention.

### 4.2. Body Composition of Athletes

Body composition results suggest that athletes competing in sports where low body weight is emphasized (lean sports), such as aesthetic and endurance sports, had lower PBFs. This aligns with the classification of lean and non-lean sports by Mancine et al. (2020) [8] and underscores the physical demands and aesthetic expectations that drive athletes in these disciplines toward lower body weight and fat. Interestingly, no significant relationship was observed between PBF and DE risk scores (DESA-6 and EAT-26) in our sample. This contrasts with Magee et al. (2023), who found a negative correlation between low BF and ED risk, particularly in female athletes [39]. Our findings suggest that, within this sample, DE risk may be more influenced by psychological and behavioral factors than by body composition alone. Future research could further explore this complex relationship, potentially examining the roles of psychological factors and the individual’s experience of pressure related to body composition.

### 4.3. Nutrition Knowledge of Athletes

Our findings indicate that female athletes scored higher on both general and sports nutrition knowledge questions (as assessed by A-NSKQ), with no significant differences between the study groups. These findings align with previous studies suggesting that female athletes often exhibit greater nutrition awareness than their male counterparts [37,38,39]. This discrepancy highlights the need for future studies to explore how knowledge is applied in athletes’ dietary practices and whether specific educational interventions can effectively reduce DE risk in high-risk sports. It may also be interesting and beneficial to explore possible differences between sport disciplines, an approach which was not possible in this study due to the heterogeneity of the sample. Concerning this present study, it is also notable that, except for nutrition knowledge, males had preferable (less risky) results in the assessments.

Limited information exists on the disordered eating risk, body composition, and nutrition knowledge of Hungarian elite athletes. This study addresses this gap, providing valuable insights and paving the way for future research in this area. The results suggest that DE risk in athletes is multifactorial, influenced by a combination of sport-specific demands (appearance or weight), body composition expectations for better athletic performance, and, potentially, an inadequate level of nutrition knowledge. Matching this complexity, our results also suggest that identifying athletes at risk of developing DE requires simultaneously using multiple complimentary assessment tools.

### 4.4. Comparative Sensitivity of DESA-6 and EAT-26 in Detecting Disordered Eating Risk

The novelty of our research is that we found associations with relatively newly validated questionnaires (DESA-6 and A-NSKQ, between 2018 and 2021) that have not been found in the scientific literature yet. Previous studies have reported that symptoms of DE and ED are unlikely to improve without appropriate treatment [23,24,25]; therefore, early detection is imperative. In this study, although a significant small-to-medium correlation was found between DESA-6H and EAT-26 total scores, DESA-6H identified fewer athletes as being at risk of DE compared to the EAT-26. This association is weaker than the original validation study [43], where a larger sample size and fewer sport disciplines yielded more statistically significant results. These findings highlight the importance of employing multiple validated assessment tools that account for the diversity in athlete populations [12]. However, according to a systematic review of ED screening tools for athletes, DESA-6 was found to be a suitable questionnaire based on validity, reliability, sensitivity, specificity, and receiver–operator–curve (ROC) statistics [70]. Our ROC statistics showed that DESA-6H is a questionnaire that shows high specificity and low sensitivity based on the internationally recommended cut-off point. When choosing different cut-off values, the researcher can decide what to focus on: being sensitive, so that it has a high chance of detecting potential cases (at the cost of misidentifying some athletes who are not actually at risk), or being very specific to avoid “misidentification”, but at the cost of missing real cases. The higher sensitivity cut-off point identified by us in our own sample is a potentially good basis for further testing in larger Hungarian and international samples. In line with other international research, DESA-6 was found to be a suitable tool for assessing DE risk with a considerable diagnostic accuracy [43].

It must be taken into account that the development of DE and ED is influenced by complex, sport-specific pressures around body image and performance [71]. In another study measuring an extended Hungarian sample of elite (*n* = 91) and recreational athletes (*n* = 90), correlations of DESA-6H with EAT-26 and EAT-26 subscales showed quite similar results, as there was a significant medium correlation between DESA-6H and the EAT-26 total score, DESA-6H and EAT-26 dieting, and DESA-6H and the bulimia subscale [44]. When comparing these results across gender groups, women scored higher (and had less favorable results) compared to men, with a significant effect size in relation to the EAT-26 total score and the dieting scale. The results by nutritional status showed that, across most variables, normal-weight individuals had less worrisome results compared to their overweight and obese counterparts, with a significant effect size in the case of DESA-6H. The most prominent result was obtained from the comparison of DESA-6H scores between the healthy and the overweight and obese groups, with a very large effect size, which is also the most meaningful result of this present study.

### 4.5. Limitations and Future Directions

Whilst this study provides important insights into disordered eating (DE) risk, body composition, and nutrition knowledge among elite Hungarian athletes, several limitations should be acknowledged. Firstly, the sample size was relatively small, and, while the study included athletes from diverse sport disciplines, the distribution was uneven across categories and did not cover the full spectrum of sport disciplines. Also, the relatively small sample size did not allow for granularity at the sport discipline or sport group (e.g., power, endurance, skill-based, and team sports) level. This limited sample diversity may have restricted the generalizability of the findings to other athlete populations, especially those outside of Hungary or in sports not represented in our sample. Future research could benefit from larger, more diverse samples to better capture variations in DE risk and nutrition knowledge across diverse cultural contexts. When mentioning the sample size, a problem with the response rate occurred which had been previously mentioned over NSKQ and A-NSKQ development [45,46]. In our study, 16 responses were omitted due to age. Additionally, our reliance on self-report questionnaires, including the DESA-6H, EAT-26, and A-NSKQ, may have introduced response bias, as participants might underreport or misinterpret certain behaviors and knowledge levels. Incorporating clinical interviews or objective measures of dietary intake and eating behaviors could help verify and expand upon these self-reported data [12,72]. Results from DESA-6H and the general nutrition knowledge scale of A-NKSQ should be interpreted with caution due to their less than satisfactory internal consistency reliability.

Further, although body composition measurements were included, we did not assess all physiological indicators of low energy availability (LEA), such as hormonal profiles, which are valuable for understanding the full scope of DE’s impacts on athletes’ health. Petrie and Greenleaf emphasize that the prevalence of eating disorders in athletes is greatly influenced by the methods and the sample selection used [69]. Therefore, it is crucial to conduct additional studies similar to previous ones, which also examined hormonal profiles, serum iron levels, and other metabolic parameters, to better assess the risk of DE and ED [73,74,75]. Future studies may integrate additional physiological assessments (e.g., blood test, body composition measurements, etc.) to provide a more comprehensive overview of DE-related health consequences. Based on our findings and previous research, the EAT-26 and DESA-6 questionnaires are effective screening tools and can form a reliable part of the diagnostic process for assessing ED and DE. Recent studies have also shown that peculiar chest wall conformations may also play a role in the development of ED [76], a factor which we did not take into account. This evidence indicates that individuals with a concave-shaped chest wall conformation and/or pectus excavatum have an increased prevalence of mitral valve prolapse and anxiety disorders [77]; thus, among athletes, those with a concave-shaped chest wall conformation might have an increased prevalence of anxiety and concomitant DE/ED in comparison with those having a more spheroidal chest shape. Incorporating this variable in future studies could enhance the precision of identifying individuals at risk of DE/ED.

Our study also highlights a need to monitor the development of DE risk, as well as exploring the long-term effects of DE risk on athletes’ health and performance. The cross-sectional design does not allow for these. Future research should employ longitudinal designs to monitor athletes over time, examining whether initial DE risk scores predict a later development of eating disorders (ED), health complications, or performance outcomes. Because our findings suggest gaps in nutrition knowledge among athletes, future studies should also examine the impact of targeted nutrition education programs on DE risk and overall athlete well-being, exploring whether improved nutrition knowledge directly mitigates DE behaviors and enhances health and performance in elite sports contexts.

It is crucial to start learning about adequate nutrition as early as possible; thus, in case of junior athletes, involving parents can also increase effectiveness. This may contribute to avoiding/reducing the development of nutrition-related problems (mainly DE and ED). The support of athletes is important not only for sports performance, but also for long-term health preservation. The results, so far, show that knowledge does not reach the acceptable level, and the risk of DE and ED is also present; therefore, urgent intervention is needed which aims to increase athletes’ nutrition knowledge and prevent DE and ED. The sooner we provide appropriate care to those who are already at risk, the greater the chance of reversing the condition.

Athletes and their entourage (e.g., team doctor, nutritionist, psychologist, coach, etc.) should work together to find a balance between the desired bodyweight for optimal athletic performance and health. In doing so, sport nutritionists and dietitians should prioritize tailored nutritional education and support for athletes, especially for those in high-risk sports. Coaches and athletic trainers should also be aware of these risks to better monitor and guide their athletes, helping foster healthier attitudes and behaviors around food and body composition. Sports governing bodies and healthcare providers working with athletes should consider incorporating regular DE risk assessments and nutrition knowledge training as part of standard athletic care.

## 5. Conclusions

The results highlight the nuanced health risks associated with disordered eating (DE) and the gaps in nutrition knowledge among elite athletes. Showing that female athletes, especially those in endurance and weight-dependent sports, are more vulnerable to DE underscores the potential impact of these risks on athletic performance, overall health, and long-term well-being. The low observed nutrition knowledge levels indicate that many athletes may lack the dietary insights necessary to support optimal training and recovery, such knowledge being crucial for both performance and health maintenance. Early intervention and education initiatives to prevent DE and encourage informed nutrition choices among athletes should address these specific risk factors and knowledge gaps in a pragmatic, performance-focused way. Athletes themselves may also benefit from understanding these risks, encouraging them to seek professional nutritional guidance early in their careers to avoid developing potential health-compromising practices which may also negatively impact their athletic performance.

Our findings highlight the importance of early detection and sport nutrition education for all elite athletes, with a particular focus on female athletes in endurance and weight-dependent sports. Further research is needed to refine screening tools like the EAT-26 and DESA-6H for use in elite athletes, including developing a more nuanced approach to cut-off points, tailored for early risk identification to support prevention efforts or for a timely diagnosis to improve the management of DE and ED.

## Figures and Tables

**Table 1:** Correlations between DESA-6H and EAT-26 scores (Kendall’s correlation coefficients).

Variables	1.	2.	3.	4.	5.	6.
1. DESA-6H	—					
2. EAT-26	0.237 *	—				
3. BMI	0.261 *	0.059	—			
4. PBF	0.114	0.076	0.292 **	—		
5. GENNUT	0.127	0.223 *	−0.138	−0.107	—	
6. SPORTNUT	0.246 *	0.258 *	−0.05	−0.032	0.501 **	—
7. ANSKQ S	0.259 *	0.259 *	−0.061	−0.069	0.701 **	0.854 **

DESA-6H = DESA-6H scores, EAT-26 = EAT26 total scores, BMI = body mass index, PBF = percent body fat, GENNUT = A-NSKQ general nutrition knowledge, SPORTNUT = A-NSKQ sport nutrition knowledge, ANSKQ S = A-NSKQ total score, * *p* < 0.05, ** *p* < 0.01.

**Table 2:** Differences between DESA-6H and EAT-26 results by gender.

Variables	Males (*n* = 28)		Females (*n* = 43)		*U*	*Z*	*p*	Cohen-*d*
	M	SD	M	SD				
DESA-6H	0.50	1.10	0.80	1.9	591.5	−0.127	0.899	0.184
EAT-26	8.50	4.20	12.9	8.00	403.00	−2.349	0.019	0.65

*n* = number of participants, M = mean, SD = standard deviation, *p* = significance level, Cohen-*d* = effect size indicator, DESA-6H = DESA-6H scores, EAT-26 = EAT-26 total scores.

**Table 3:** Differences between EAT-26 and DESA-6H scores based on nutritional status.

Variables	Normal (*n* = 53)		Overweight and Obese (*n* = 15)		*U*	*Z*	*p*	Cohen-*d*
	M	SD	M	SD				
DESA-6H	0.46	1.53	1.73	1.75	211.00	−2.832	0.005	1.361
EAT-26	10.71	7.18	12.2	7.01	343.50	−0.801	0.423	0.209

*n* = number of participants, M = mean, SD = standard deviation, *p* = significance level, Cohen-*d* = effect size indicator, DESA-6H = DESA-6H scores, EAT-26 = EAT-26 total scores.

**Table 4:** Differences in nutrition knowledge scores between at-risk and non-risk athletes based on DESA-6H.

Variables	Non-Risk Across DESA-6H (*n* = 62)		At-Risk Across DESA-6H (*n* = 9)		*U*	*Z*	*p*	Cohen-*d*
	M	SD	M	SD				
GENNUT	6.11	2.14	6.88	1.91	247.00	−0.560	0.575	0.364
SPORTNUT	9.85	3.91	11.77	2.16	189.50	−1.555	0.120	0.512
ANSKQ S	15.96	5.49	18.66	3.74	197.50	−1.412	0.158	0.508

GENNUT = A-NSKQ general nutrition knowledge, SPORTNUT = A-NSKQ sport nutrition knowledge, ANSKQ S = A-NSKQ total score.

**Table 5:** Differences in nutrition knowledge between at-risk and non-risk athletes based on EAT-26 scores.

Variables	Non-Risk Across EAT-26 (*n* = 47)		At-Risk Across EAT-26 (*n* = 24)		*U*	*Z*	*p*	Cohen-*d*
	M	SD	M	SD				
GENNUT	5.96	2.24	6.83	1.78	540.00	−1.404	0.160	0.415
SPORTNUT	9.55	4.04	11.37	2.94	366.00	−2.419	0.016	0.491
ANSKQ S	15.51	5.74	18.21	4.21	381.50	−2.224	0.026	0.511

*n* = number of participants, M = mean, SD = standard deviation, *p* = significance level, Cohen-*d* = effect size indicator, GENNUT = A-NSKQ general nutrition knowledge, SPORTNUT = A-NSKQ sport nutrition knowledge, ANSKQ S = A-NSKQ total score.

**Table 6:** DESA-6H accuracy measures of the DESA-6H against EAT-26 as the ‘gold standard’ measure.

	DESA-6H Cut-Off *J*		Total	DESA-6H Cut-Off Int		Total
	Non-Risk	At-Risk		Non-Risk	At-Risk	
EAT-26 non-risk	TN 33	FP 14	47	TN 45	FP 2	47
EAT-26 at-risk	FN 4	TP 20	24	FN 17	TP 7	24
Total	37	34	71	62	9	71
Sensitivity	83.3%			29.1%		
Specificity	70.2%			95.7%		
LR+	2.79			6.77		
LR-	0.36			0.15		
Accuracy	74.6%			73.2%		

cut-off *J* = DESA-6Hmax cut-off to be, cut-off int = DESA-6H cut-off of international norm, TN = true negative, TP = true positive, FN = false negative, FP = false positive, LR+ = positive likelihood ratio, LR- = negative likelihood ratio.

## Data Availability

All data are included in the study.

## Abbreviations

AUC	Area under the curve
DE	Disordered eating
ED	Eating disorder
EDI	Eating Disorder Inventory
DSM-V	Diagnostic and statistical manual of mental disorders
BN	Bulimia nervosa
BED	Binge eating disorder
BDD	Body dysmorphic disorder
LEA	Low energy availability
BF	Body fat
IC	Internal consistency
A-NSKQ	Abridged nutrition for sport knowledge questionnaire
KR-20	Kuder–Richardson formula 20
DESA-6	Disordered Eating Screen for Athletes
EAT-26	Eating Attitude Test
BMI	Body Mass Index
DESA-6H	Disordered Eating Screen for Athletes—Hungarian version
IOC	International Olympic Committee
APA	American Psychiatric Association
PBF	Percent body fat
NSKQ	Nutrition for Sport Knowledge Questionnaire
WHO	World Health Organization
ACE	American Council of Exercise
EAT-26	S EAT-26 total score
EAT-26	D EAT-26 dieting behavior subscale
EAT-26	O EAT-26 oral control subscale
EAT-26	B EAT-26 bulimia and food preoccupation subscale
GENNUT	General nutrition knowledge
ROC	Receiver operating characteristic
SPORTNUT	Sport nutrition knowledge
ANSKQ S	A-NSKQ total score
